# Diabetic retinopathy incidence, predictors and its association with longitudinal fasting blood sugar level changes among diabetes mellitus patients in Ethiopia: joint model

**DOI:** 10.3389/fendo.2024.1363757

**Published:** 2024-07-08

**Authors:** Habtamu Wagnew Abuhay, Ayenew Molla Lakew, Haileab Fekadu Wolde, Berhanu Mengistu, Mandefro Tadesse Legesse, Melaku Kindie Yenit

**Affiliations:** ^1^ Department of Epidemiology and Biostatistics, Institute of Public Health, College of Medicine and Health Sciences, University of Gondar, Gondar, Ethiopia; ^2^ Department of Human Nutrition, Institute of Public Health, College of Medicine and Health Sciences, University of Gondar, Gondar, Ethiopia; ^3^ College of Medicine and Health Sciences, University of Gondar, Gondar, Ethiopia; ^4^ School of Health and Medical Sciences, Centre for Health Research, University of Southern Queensland, Ipswich, QLD, Australia

**Keywords:** incidence, diabetic retinopathy, fasting blood sugar, diabetic mellitus, joint modeling, Ethiopia

## Abstract

**Background:**

Diabetes mellitus (DM) is a global public health problem characterized by an elevated blood glucose level. Monitoring blood sugar levels is vital for effective diabetes management and preventing complications. However, the association between longitudinal biomarkers and the incidence of diabetic complications is often overlooked. Therefore, this study aimed to assess the incidence of diabetic retinopathy, predictors, and association with longitudinal fasting blood sugar level changes among diabetes mellitus patients in Ethiopia.

**Methods:**

A multicenter retrospective follow-up study was carried out in referral hospitals in Amhara region, Ethiopia. A random sample of 462 newly diagnosed DM patients was selected. The proportional hazard assumption was checked for the survival sub-model, and for the longitudinal sub-model, the normality assumption was checked. Then the joint modeling with time-dependent lagged parameterizations was fitted. Model assumptions and comparisons were checked. Finally, the hazard ratio with a 95% confidence interval (CI) with a corresponding P-value<0.05 was used to identify predictors.

**Results:**

In this study, Overall, 54 patients developed DR, and the incidence rate was 2.33 per 1000 person-months over the follow-up period, with a 95% CI of [1.78, 3.05]. Rural residence (AHR = 2.21, 95% CI: [1.21, 4.05]), hypertension co-morbidity (AHR = 3.01, 95% CI: [1.85, 6.53]), and longer duration of DM (>5 years) (AHR = 2.28, 95% CI: [1.91, 5.15]) were important predictors for the incidence of DR. In addition, the incidence of DR was substantially correlated with the time-dependent lagged value of FBS change (AHR = 4.20, 95% CI [1.62, 10.85]).

**Conclusions:**

In this study, the incidence of diabetic retinopathy was somewhat high when compared to prior similar studies in Ethiopia. A joint model of longitudinal fasting blood sugar level changes was significantly associated with an increased risk of DR. Besides, being rural residence, hypertension co-morbidity, and a longer duration of DM were significant predictors for the incidence of DR. Therefore, public awareness, an integrated care approach, and prioritizing glycemic control are highly recommended.

## Introduction

Diabetes mellitus is a prevalent global public health issue, affecting millions of people worldwide (1). In 2021, approximately 537 million individuals had diabetes worldwide (2). Diabetes disproportionately affects the global south country (3). The African region is particularly affected, with an estimated 23.6 million adults living with diabetes. However, the prevalence of diabetes varies between countries in the region (4). Ethiopia, a global south country, ranks among the leading five African nations with a substantial number of diabetic patients, with around 1.92 million in 2021 (5).

Uncontrolled diabetes mellitus, characterized by elevated blood glucose levels, can severely affect overall health (6). If left unmanaged, it can lead to macro- or microvascular complications affecting multiple systems in the body, such as diabetic retinopathy and others (2, 7). Diabetic retinopathy, which is characterized by damage to the blood vessels in the retina (8), is one of the most prevalent micro-vascular complications and the leading cause of vision loss among diabetes patients (9, 10). Additionally, uncontrolled diabetes can have detrimental effects on other organs and systems, increasing the risk of heart disease, stroke, kidney failure, neuropathy, and lower limb amputations, thereby impacting individuals’ quality of life and resulting in increased healthcare costs and socioeconomic burdens (7, 11).

Approximately one-third of all individuals with diabetes worldwide have some degree of diabetic retinopathy (12). In Africa, the prevalence of diabetic retinopathy ranges from 7.0 to 62.4% (13). In Ethiopia, the prevalence of DR is increasing, with reports indicating that 34.1% of diabetes patients in the country have some form of DR (8).

The development of diabetic retinopathy among diabetes patients is closely associated with uncontrolled blood glucose levels (14). Over time, persistently elevated blood sugar levels can damage the small blood vessels in the retina, leading to the onset and progression of diabetic retinopathy (15). Proper management of blood glucose levels is crucial to reducing the risk of developing diabetic retinopathy (16, 17). Numerous factors, including duration of diabetes, type of diabetes, body mass index, and comorbidity, impact the incidence of diabetic retinopathy (18–21).

Fasting blood sugar tests are essential predictors for diabetes diagnosis and management, as they can help prevent complications (22). However, in resource-limited settings like Ethiopia, the longitudinal association between clinical biomarkers and the incidence of diabetic retinopathy among diabetes patients is rarely considered.

Therefore, this study aims to assess the incidence and predictors of diabetic retinopathy and their association with longitudinal changes in FBS levels among diabetic mellitus patients. Estimating the incidence of diabetic retinopathy provides insights for implementing effective strategies to reduce diabetic-related complications. Joint modeling analysis enables a comprehensive understanding of the relationship between blood glucose levels and the incidence of diabetic retinopathy, helping identify high-risk individuals for targeted prevention and improved management of diabetes mellitus.

## Methods

### Study design, period, and settings

A multi-centered and institutionally based retrospective follow-up study was carried out between January 1, 2011, and December 30, 2021. The University of Gondar, Felege Hiwot, and Debre Tabor Compressive Referral Hospitals in the Amhara region of Ethiopia were the three referral hospitals where the study was carried out.

### Sample and population

The study included newly diagnosed diabetes patients aged 15 or older who received follow-up care at selected referral hospitals between January 1, 2011, and December 30, 2021. A total of 462 study subjects were estimated. For the survival parts, the sample size was determined using factors significantly associated with the incidence of DR from previous studies, and the sample size was calculated using the Schoenfeld formula (23).


$$\text{E}:\frac{{\left(\frac{\text{Za}}{2}+\text{Zβ}\right)}^{2}}{\text{P}1\text{P}2{\left(\text{lnHR}\right)}^{2}}\;\text{AND n}=\frac{E}{\text{P}\left(\text{E}\right)}$$

Where E= number of required events, n= sample size, HR is the hazard ratio of selected covariates, p1 proportion of subjects under the exposure group, p2 = 1 – p1, and P(E) is the probability of an event.

Therefore, based on the results we found from previous retrospective follow-up studies done in Ethiopia, being male associated with diabetes retinopathy (AHR =1.94) (20), and assuming the following assumptions: power = 80%, ᾳ = 0.05, ß = 0.2, the sample size becomes 280 under this approach.

For the longitudinal part, the sample size was determined using the Diggle formula taken for repeated measurements for longitudinal parts (24).


$$\text{N}=\frac{4{\left(Z\frac{a}{2}+Z\text{β}\right)}^{2}\alpha^{2}\;(1+(m-1)p)}{md^{2}}$$

*N*: is the total sample size, *d* is the effect size, *m* is the number of time points repeated measurement, *ρ* is a correlation between repeated measurements and *σ* ^2^ is the variance of outcome variables. Assuming a significance level of 0.05, power of 0.8, *ρ* = 0.5, and effect size of 0.8, *m* = 8 times points based on a previous study, From a study conducted in Jimma on longitudinal FBS change we have, *σ*^2 =4.9 (random intercept model) (25). Using z_α/2_ = 1.96, Zβ = 0.842, and inserting all quantities in the formula.


$$N=\frac{4{\left(1.96+0.842\right)}^{2}4.9(1+(8-1)0.5)}{8*0.8*0.8}=136$$

Therefore, the final sample size for this study was 280, then after adding 10% incompleteness and considering design effect 1.5. The final sample size was 462 DM patients.

A computer-generated simple random sampling technique was employed to select records from the newly diagnosed diabetes patients. DM Patients with uncertain dates of enrollment, as well as those who had diabetic retinopathy at the time the research started, were excluded.

### Study variables, data collection tools, and procedures

Based on previously published research and the medical records of patients with diabetes mellitus (DM), a structured data collection checklist has been developed for this study. The checklist consisted of four parts, each covering different aspects of the patient’s characteristics.

The first part of the checklist focused on socio-demographic characteristics, including variables such as the age of study participants, sex, and residence. This information provided insights into the demographic profile of the study participants. The second part of the checklist captured clinical characteristics, encompassing factors such as body mass index (BMI), type of treatment, family history of DM, duration of DM, type of DM, presence of diabetic nephropathy, hypertension, diabetic neuropathy, peripheral arterial disease, and history of stroke. The third part of the checklist encompassed physiological characteristics, including measurements of high-density lipoprotein (HDL), low-density lipoprotein (LDL), creatinine levels, triglyceride levels, cholesterol levels, protein urea, systolic blood pressure (mmHg), and diastolic blood pressure (mmHg). Lastly, the fourth part of the checklist focused on capturing fasting blood sugar levels (FBS), which were measured repeatedly throughout the study. FBS levels were an important indicator for assessing the patient’s glycemic control.

In this study, the dependent variable was diabetic retinopathy, defined as the presence of at least one micro-aneurysm in any field, the presence of hemorrhages, or the occurrence of maculopathy in individuals with diabetes mellitus (26). The determination of whether a patient had diabetic retinopathy or not was made by reviewing their medical documents.

### Data processing, and analysis

For this study, version 4.4 of EpiData was used to enter the data, while R version 4.3 was used for analysis. To prepare the data for analysis, the data was combined and coded as required. Descriptive statistics were computed for both categorical and continuous variables to summarize the data. Individual and average profile plots were created for the longitudinal analysis to visualize the trends over time. Model comparison was conducted using AIC (Akaike Information Criterion) values to select the best-fitting longitudinal sub-model.

Additionally, the normality assumption of the linear mixed effect model was assessed for fasting blood sugar (FBS) values. In the survival analysis, the incidence of diabetic retinopathy was estimated using Kaplan-Meier (KM) curves, and the log-rank test was employed to compare survival times between different variables. Before fitting the survival sub-models, proportional hazard assumptions (PHA) were investigated.

The relationship between changes in longitudinal FBS and diabetic retinopathy was evaluated using the alpha value obtained from the employed joint model. A variable was taken to be significant in the multivariable joint model if its p-value was less than 0.05 and it was within the 95% confidence interval.

## Ethical approval and consent to participate

This study received ethical approval from the University of Gondar, College of Medicine and Health Science, Institute of Public Health, Institutional Review Board (IRB) with reference number Ref No/IPH/1397/2020. Official permission was also obtained from the clinical directors of the selected referral hospitals. The study adhered to relevant guidelines and regulations. Informed consent was waived by the Institutional Review Board due to the retrospective nature of the study, and the data were anonymized and kept confidential.

## Results

### Sociodemographic and clinical related characteristics

A total of 462 patients with newly diagnosed DM were included in this study. The study participants had a mean age of 45.78 (SD ±15.92) years. Most of the study participants, 257 (55.6%) and 238 (51.5%), were male and urban residents. Almost two-thirds of the study’s participants, 290 (62.7%), had type two diabetes mellitus (T2DM), and one-third of them, 134 (29.0%), had hypertension. Most of the 220 (47.6%) patients enrolled in this study were on oral hypoglycemic agent (OHA) treatment (**Table 1**).

**Table 1:** Sociodemographic and clinical characteristics of DM patients on treatment in the Amhara region of Ethiopia.

Variables	Categories	Frequency	Percent (%)
Sex	Female	205	44.37
	Male	257	55.63
Residence	Urban	238	51.52
	Rural	224	48.48
DM Family History	Yes	99	21.43
	No	284	61.47
	Unknown	79	17.10
DM’s duration	< 5 year	378	81.81
	≥ 5 year	84	18.19
Type of treatment	OHA	220	47.62
	Insulin	198	42.86
	Both	44	9.52
Type of DM	T1DM	172	37.23
	T2DM	290	62.77
Hypertension	Yes	31	6.71
	No	431	93.29
Patients last status	Alive	382	82.7
	Lost follow-up	20	4.32
	Died	6	1.29
	Event	54	11.69
Referral hospital	UoGCSH	213	46.1
	FHCSH	173	37.45
	DTCRH	76	16.45

OHA, Oral hypoglycemic agent; T1DM, Type one diabetic mellitus; T2DM, Type two diabetic mellitus; UoGCSH, University of Gondar Compressive Specialized Referral Hospital; FHCSH, Felege Hiwot Compressive Specialized Hospital; DTCRH, Debre Tabor compressive referral hospital.

### Physiological characteristics, and diabetic related complication

Among the patients, 10.9% had elevated triglyceride levels, while 13.7% had borderline total cholesterol levels. A majority of the patients (58.5%) had high-density lipoprotein cholesterol (HDL-C) levels above 40mg/dl, and 6.9% DM patients had diabetic nephropathy, as shown in (**Table 2**).

**Table 2:** Physiologic characteristics, and diabetic-related complications among diabetic patients at treatment in Amhara region, Ethiopia.

Variables	Categories	Frequency	Percent (%)
Triglyceride(md/dl)	<150	139	72.77
	150 -199	31	16.24
	≥200	21	10.99
Total cholesterol(mg/dl)	<200	163	83.16
	200 -239	27	13.78
	≥240	6	3.06
LDL-C(md/dl)	<100	159	80.71
	≥100	38	19.29
HDL-C(md/dl)	<40	79	41.15
	≥40	113	58.85
SBP(mmHG)	<140	331	71.65
	≥140	131	28.35
DBP (mmHG)	<90	400	86.58
	≥90	62	13.42
Neuropathy	Yes	38	8.23
	No	424	91.77
Nephropathy	Yes	32	6.93
	No	430	93.07
Peripheral arterial disease	Yes	23	4.98
	No	439	95.02

md/dl, Milligram per deciliter; HDL-C, High-density lipoprotein creatinine; LDL -C, Low-density lipoprotein creatinine; SBP, Systolic blood pressure; DBP, Diastolic blood pressure; mmHG, Millimetre of mercury.

### Incidence of diabetic retinopathy

**Figure 1:**
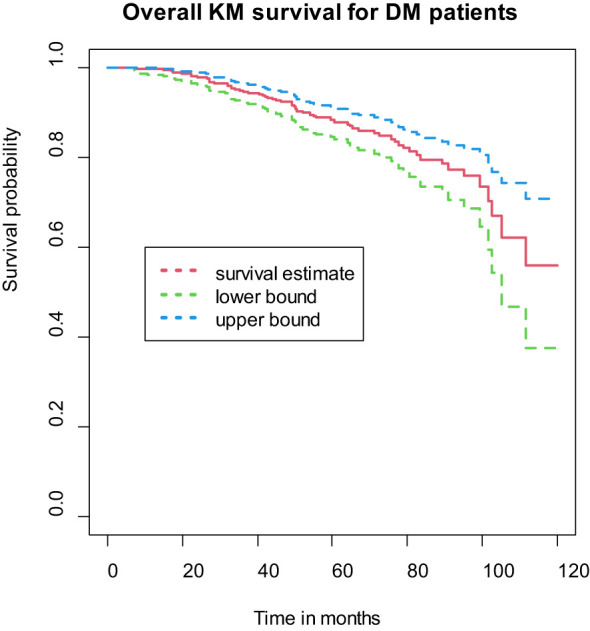
Overall Kaplan-Meier (KM) of survival curves for DM patients on treatment in Ethiopia.

The study followed a cohort of diabetes patients ranging from 6 to 120 months, with a median follow-up time of 46 months (interquartile range: 29 to 71 months). Among the 462 study participants, a total of 54 individuals (11.69%, 95% CI [8.74, 14.62]) developed diabetic retinopathy throughout 23,111.0 person-months (PM) of observation. The calculated incidence density was 2.33 per 1000 PM (95% CI: [1.78, 3.05]), or 2.97 per 100 person-years (95% CI: [2.26, 3.91]), as depicted in (**Figure 1**).

### Exploring fasting blood sugar change over time

#### Individual FBS profile of DM patients

**Figure 2:**
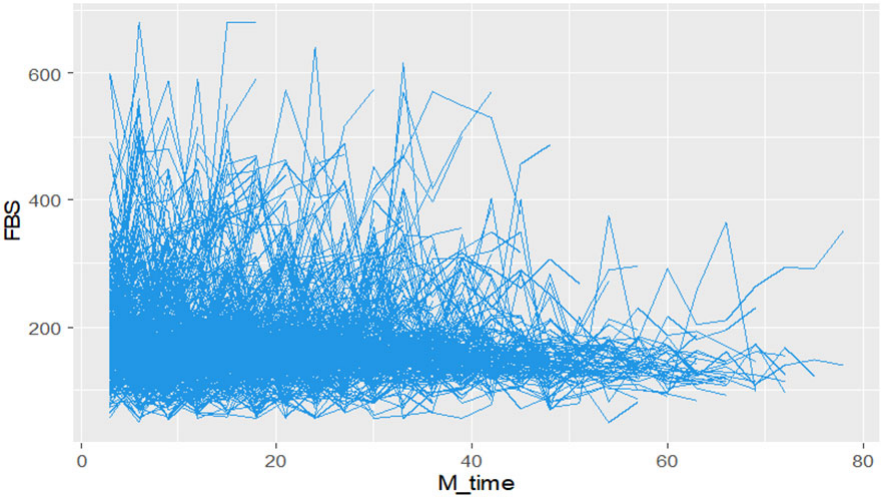
Individual profile plots Fasting Blood Sugar (FBS) over time for DM patients on treatment in Ethiopia.

**Figure 3:**
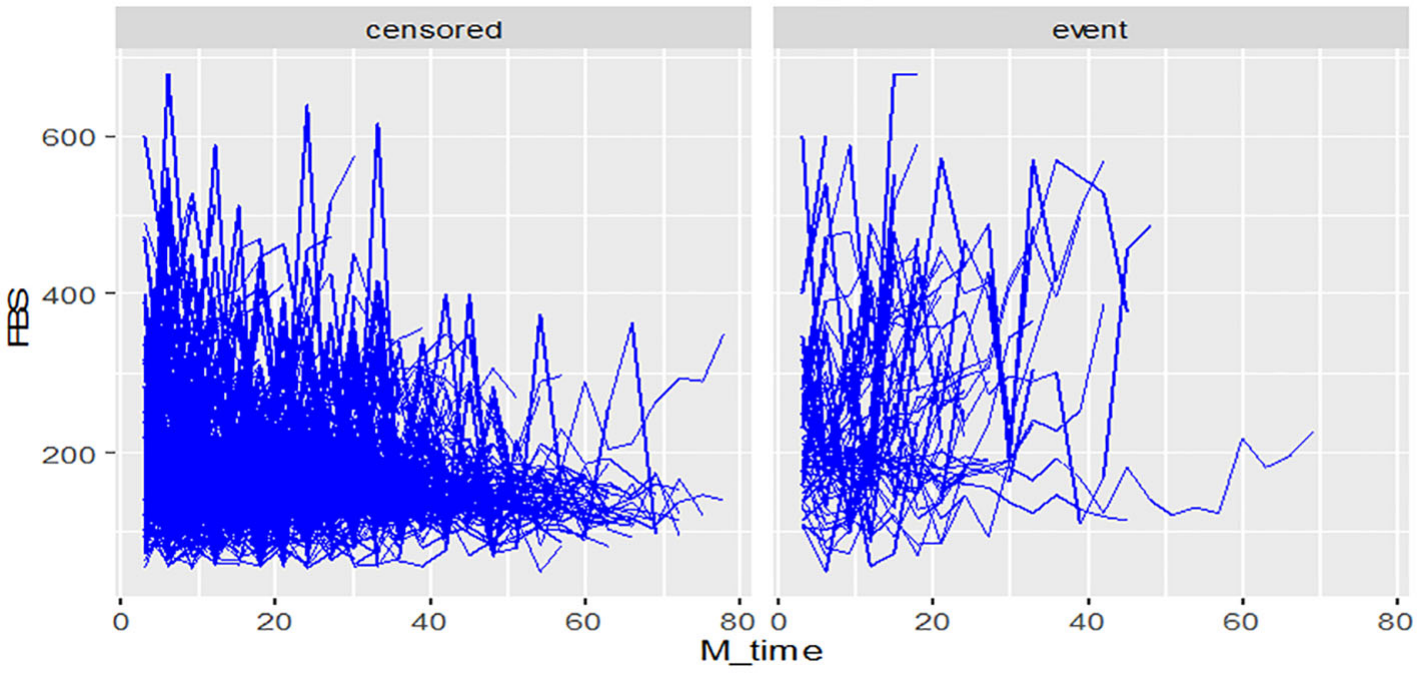
Individual profile plots by status of diabetic retinopathy for DM patients on treatment in Ethiopia.

During the study period, diabetes patients on treatment underwent a maximum of twenty-eight and a minimum of three fasting blood sugar (FBS) measurements. The individual profile plots provided a comprehensive visualization of the FBS trends for each patient, revealing significant variability both within and between patients (**Figure 2**). Furthermore, the analysis of this study revealed that the subject variability among diabetes mellitus (DM) patients with events was higher compared to the censored parts. This observation provides evidence for the suitability of applying joint modeling techniques to this dataset (**Figure 3**).

#### Mean FBS profile of DM patients

**Figure 4:**
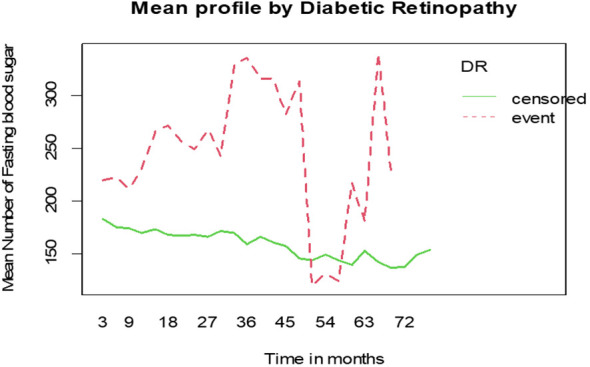
Mean profile of Fasting Blood Sugar (FBS) by status of diabetic retinopathy for DM patients on treatment in Ethiopia.

The mean of FBS among DM patients shows that individuals who developed diabetic retinopathy had higher FBS levels compared to those who did not develop diabetic retinopathy (**Figure 4**).

### Predictors of incidence of diabetic retinopathy

Joint modeling indicates the associated factors for both changes in FBS and the incidence of diabetic retinopathy. The results indicated a significant association between the 3-month lagged value of FBS and the risk of DR. The results from (**Table 3**), shows that there is a significant association (α = 1.43, p-value 0.003) between the longitudinal change in fasting blood sugar levels (log of FBS) and the risk of DR. This finding suggests that a unit increase in the past 3-month value of the log of FBS corresponds to a 4.2-fold increase in the current risk of developing DR. In addition, being a rural resident, having hypertension (comorbidity), and having a duration of diabetes greater than five years were significant predictors for the incidence of diabetic retinopathy.

**Table 3:** Joint modeling of the survival and longitudinal sub-models for diabetic mellitus patients on treatment in Amhara region referral hospitals, Ethiopia.

Variables	Categories	Diabetic Retinopathy		AHR [95% Cl]	P-value
		Censored	Event		
Sex	Male	222	35	1	
	Female	186	19	0.77 [0.43,1.39]	0.403
Residence	Urban	220	18	1	
	Rural	188	36	**2.21 [1.21, 4.05]**	0.009**
Family history of DM	No	321	42	1	
	Yes	87	12	0.57 [0.28, 1.15]	0.117
Protein urea	Negative	319	34	1	
	Positive	47	19	1.19 [0.64, 2.20]	0.578
Duration DM	< 5 year	350	28	1	
	≥5 year	58	26	**2.28 [1.91, 5.15]**	0.002**
Neuropathy	No	386	38	1	
	Yes	22	16	1.73 [0.83, 3.59]	0.138
Nephropathy	No	297	31	1	
	Yes	111	23	0.71 [0.38, 1.32]	0.279
Hypertension (co-morbidity)	No	389	42	1	
	Yes	19	12	**3.01 [1.85, 6.53]**	0.005**
Association parameter (lag =3 months)				**4.20 [1.62, 10.85]**	0.003**

DM, Diabetic mellitus, **p-value<0.01, *p-value< 0.05. Bold values indicate variables that have a significant associated with the incidence of diabetic retinopathy.

After controlling for other variables in the model, rural patients had a hazard of developing diabetic retinopathy (DR) that was 2.21 times higher compared to urban patients (adjusted hazard ratio [AHR] = 2.21, 95% CI: [1.21, 4.05]). DM patients with hypertension (co-morbidity) had a hazard of developing DR that was 3.01 times higher than those without hypertension (co-morbidity) when accounting for other variables (AHR = 3.01, 95% CI: [1.85, 6.53]). Furthermore, after controlling other factors in the model, DM patients with a diabetes duration greater than five years had a hazard of experiencing DR that was 2.28 times higher compared to those with a duration of less than five years (AHR = 2.28, 95% CI: [1.91, 5.15]). These findings are presented in (**Table 3**).

## Discussion

Diabetic retinopathy (DR) is a significant global public health concern and a leading cause of blindness. Understanding the incidence of DR and identifying its predictors are vital for effective prevention and management strategies. Furthermore, investigating the impact of fasting blood sugar (FBS) changes on the risk of DR can provide valuable insights into the disease progression. This study aimed to determine the incidence of DR, identify its predictors, and examine the association between longitudinal FBS trajectory and the incidence of DR among newly diagnosed diabetes patients on treatment in referral hospitals in the Amhara region, Ethiopia. By utilizing a joint modeling approach, the researchers could simultaneously analyze the longitudinal FBS data and the occurrence of DR to assess their association.

In this study, the incidence of diabetic retinopathy was observed in 11.69% of the participants, with an incidence density of 2.9 per 100 person-years (PY) of observation. This finding aligns with a previous study in central Ethiopia (20). However, it is noteworthy that the incidence reported in this study is higher compared to studies conducted in northwest Ethiopia (26), China (27), and Bangladesh (28), where the incidence of diabetic retinopathy was 2.0, 1.81 and 1.75 per 100 PY of observation, respectively. This discrepancy in incidence rates could be attributed to several factors. Firstly, variations in the study population, such as differences in lifestyle factors and comorbidities, may contribute to differences in diabetic retinopathy incidence. Additionally, variations in healthcare facilities and the quality of care provided in different settings could impact the detection and management of diabetic retinopathy, thereby influencing the reported incidence rates.

According to this finding, the risk of developing diabetic retinopathy (DR) was higher for rural patients than for urban patients. This is consistent with previous research conducted in Bangladesh (29), which demonstrated that semi-urban subjects faced a higher risk of diabetic retinopathy when compared to their urban counterparts. Additionally, a study in Beijing, China, revealed a significant association between diabetic retinopathy and patients residing in rural regions (30). This could be because rural diabetes mellitus (DM) patients may have poorer self-care practices and limited access to healthcare services, leading to suboptimal management of their diabetes and higher susceptibility to DR (31). Poor health care-seeking behaviors among rural populations, coupled with limited resources and infrastructure, can contribute to delays in diagnosis and treatment of DM-related complications, including DR. Furthermore, individuals residing in rural areas may face additional challenges, such as limited availability of specialized eye care services and reduced awareness about the importance of regular eye examinations for DM patients.

Our study found that DM patients who also have hypertension co-morbidity had an increased risk of developing Diabetic retinopathy compared with their counterparts without hypertension. This finding is supported by studies conducted in Debre Markos (32), which showed that hypertensive patients are three times more likely to develop Diabetic retinopathy compared to non-hypertensive patients. This correlation is consistent with similar studies conducted in Arbaminch General Hospital in Ethiopia (33) and Khartoum in Sudan (34). Furthermore, existing research indicates that hypertension serves as a risk factor for both the onset and progression of retinopathy (35). The heightened blood flow associated with hypertension can potentially damage the retinal capillary endothelial cells in individuals with diabetes, leading to the manifestation of retinopathy.

Diabetes patients with a duration exceeding five years were at a 2.2 times higher risk of developing Diabetic retinopathy (DR) compared to patients with a duration of DM lower than five years. This finding is in line with similar studies conducted in Debre Markos (32), Beijing (30), and Denmark (36), which all reported a significant association between longer durations of DM and the development of DR. However, a study conducted at the University of Gondar Comprehensive Specialized Hospital in Ethiopia (26) showed conflicting results, indicating a negative association between the duration of diabetes and the likelihood of developing DR. This discrepancy may be due to the reason that the study conducted in University of Gondar specialized hospital exclusively focused on type-2 diabetes patients, who may have better metabolic control and lower levels of fasting blood sugar. This disparity in findings suggests that the improved metabolic control and lower FBS levels observed in type-2 DM patients could explain the contradictory results.

In this research, we employed a time-dependent lagged value parameterization of joint modeling to investigate the association between fasting blood sugar levels and the risk of diabetic retinopathy (DR). Our finding showed a significant correlation between the fasting blood sugar levels from the past three months and the current risk of DR. Specifically, an increase of one unit in the log-transformed fasting blood sugar level over the past three months corresponded to a 4.2-fold increase in the risk of developing DR. These results are consistent with previous studies done at the Arbaminch General Hospital and in France, which demonstrated a strong relationship between elevated levels of hemoglobin A1c and fasting plasma glucose for the presence of diabetic retinopathy (33, 37). These findings suggest that higher fasting blood glucose levels, indicative of inadequate blood glucose control, may contribute to the development of diabetic retinopathy.

The findings of this study have significant clinical and public health implications. It is crucial to increase awareness and education about the risk factors associated with diabetic retinopathy (DR). Health education programs should emphasize regular screening, glycemic control, and blood pressure management to reduce the risk of DR. Implementing regular screening programs for early detection and intervention is essential. Prioritizing glycemic control through medication adherence, lifestyle changes, and regular monitoring of blood sugar levels is crucial in reducing the risk of DR.

Effective management of hypertension is also important since it is a significant predictor of DR incidence. Controlling blood pressure levels can decrease the risk of developing DR. Further research is needed to explore additional risk factors and preventive strategies for DR, including lifestyle factors. By implementing these recommendations, healthcare providers, policymakers, and patients can collaboratively reduce the burden of diabetic retinopathy and improve outcomes for individuals with diabetes.

This study has its strengths and limitations; the study may overcome previously unexplored associations between longitudinal clinical biomarkers of FBS and the incidence of DR. It has significant clinical and public health importance by providing stronger evidence for established risk factors and helps to guide clinical practice. However, the data were collected retrospectively using secondary sources, thereby being incomplete.

## Conclusions

In this study, the incidence of diabetic retinopathy was somewhat high when compared to prior similar studies in Ethiopia. A joint model of longitudinal fasting blood sugar level changes was significantly associated with an increased risk of DR. Besides, being rural residence, hypertension co-morbidity, and a longer duration of DM were significant predictors for the incidence of DR. Therefore, public awareness, an integrated care approach, and prioritizing glycemic control are highly recommended.

Based on the findings of this study, the following recommendations are made for the concerned bodies: Health professionals should give greater attention to DM patients with the identified risk factors for DR. Patients with diabetes mellitus who also have hypertension and a duration of DM greater than five years should closely monitor and control their blood glucose levels. Further studies on this topic, including behavioral factors and prospective studies, are recommended.

## Data availability statement

The original contributions presented in the study are included in the article/supplementary material. Further inquiries can be directed to the corresponding author.

## Ethics statement

Ethical approval to conduct the study was approved by ethical review board of the University of Gondar, College of Medicine and Health Science, Institute of Public Health, Institutional review board (IRB) with reference number Ref No/IPH/1397/2020. The studies were conducted in accordance with the local legislation and institutional requirements. Due to the retrospective nature of this study, based on secondary data from the patient’s medical record, the IRB waived informed consent.

## Author contributions

HA: Conceptualization, Data curation, Formal analysis, Funding acquisition, Investigation, Methodology, Project administration, Resources, Software, Supervision, Validation, Visualization, Writing – original draft, Writing – review & editing. MY: Conceptualization, Methodology, Supervision, Validation, Writing – original draft, Writing – review & editing. HW: Conceptualization, Methodology, Software, Supervision, Validation, Writing – original draft, Writing – review & editing. BM: Conceptualization, Data curation, Validation, Writing – review & editing. ML: Conceptualization, Data curation, Validation, Writing – review & editing. AL: Conceptualization, Formal analysis, Methodology, Software, Supervision, Validation, Writing – original draft, Writing – review & editing.
